# The surgical outcome of multilevel anterior cervical discectomy and fusion in myelopathic elderly and younger patients

**DOI:** 10.1038/s41598-022-08243-8

**Published:** 2022-03-16

**Authors:** Chi-An Luo, Austin Samuel Lim, Meng-Ling Lu, Ping-Yeh Chiu, Po-Liang Lai, Chi-Chien Niu

**Affiliations:** 1 Department of Orthopaedic Surgery, Spine Division, Linkou Chang Gung Memorial Hospital, No. 5, Fuxing St., Guishan Dist., Taoyuan City, 333 Taiwan, ROC; 2 Department of Orthopaedic Surgery, New Taipei Municipal Tucheng Hospital (Built and Operated By Chang Gung Medical Foundation), No. 6, Sec. 2, Jincheng Rd., Tucheng Dist., New Taipei City, 236 Taiwan, ROC; 3 Chang Gung University College of Medicine, No.259, Wenhua 1st Rd, Guishan Dist., Taoyuan City, 333 Taiwan, ROC; 4 Department of Surgery, Section of Orthopedics, Orthopedic and Spine Surgery, Metropolitan Medical Center, No.1357, Masangkay St, Santa Cruz, 1012 Manila, Metro Manila Philippines; 5 Department of Surgery, Section of Orthopedics, Orthopedic and Spine Surgery, Chinese General Hospital, No.286, Blumentritt Rd, Sampaloc, Manila, Metro Manila Philippines; 6 Department of Orthopaedic Surgery, Spine Division, Kaohsiung Chang Gung Memorial Hospital, No. 123, Dapi Rd., Niaosong Dist., Kaohsiung City, 833 Taiwan, ROC

**Keywords:** Neurodegeneration, Geriatrics, Neurosurgery

## Abstract

The elderly population has an increased risk of degenerative cervical myelopathy due to multilevel disease, causing motor and sensory dysfunctions and a poor quality of life. Multilevel anterior cervical discectomy and fusion (ACDF) is an alternative surgical treatment option, but has a perceived higher risk of complications. The goal of this study is to report the outcome. We retrospectively reviewed patients from 2006 to 2019 undergoing multilevel ACDF for degenerative cervical myelopathy and compared outcomes and complications between elder patients (aged 70 and above) and younger patients (below 70). The patients’ comorbidities, and postoperative complications, radiographic parameters such as C2–C7 Cobb angle, C2–C7 sagittal vertical axis, inter-body height of surgical levels and fusion rate were recorded. Japanese Orthopaedic Association (JOA) score and modified Odom’s score were collected. Included were 18 elderly (mean age 74, range 70–87) and 45 young patients (mean age 56, range 43–65) with a follow-up of 43.8 and 55.5 months respectively. Three-level ACDF was the most common. The ratios of ASA class III patients were 94.4% and 48.9% (p < 0.001). The Charlson comorbidity indexes were 4.3 ± 1.03 and 2.1 ± 1.11 (p < 0.001). The average lengths of hospital stays were 4.9 and 4.6 days. Eleven patients (61.1%) in the elderly group experienced at least one short-term complication, compared with 16 patients (35.6%) in the younger group (p < 0.05). The middle-term complications were comparable (22.2% and 20.0%). The JOA score, recovery rate and modified Odom score showed comparable result between groups. Despite its extensiveness, multilevel ACDF is feasible for the elder patients with good clinical outcome and fusion rate. When compared to younger cohort, there is a trend of lower preoperative JOA score and recovery rate. The short-term complication rate is higher in the elderly group.

## Introduction

There is an aging population worldwide nowadays. Degenerative cervical diseases including degenerative disc disease, osteophyte formation and advanced spondylosis that cause cord compression at one or multiple levels of the cervical spine, become more common with age^1,2^. This compression then leads to radiculopathy by root compression, or deterioration of the spinal cord leading to degenerative cervical myelopathy (DCM). Special attention should be paid to the outcomes of disability and impaired quality of life that DCM brought to the elderly population^3^. This growing geriatric group of patients could otherwise lead an active life into their eighth decade or more. The 2020 data of the Ministry of the Interior in Taiwan show that the average lifespan was 81.32 years (male 78.11 years; female 84.75). The life expectancies of 70-year-old male and female were 85.17 and 88.50 years respectively^4^.

Anterior cervical decompression and fusion (ACDF) is the one of the most commonly performed procedures for cervical spondylosis^2,5^. Multilevel ACDF possesses the advantage of less neck pain than posterior approach^2^, and is good for patients with cervical kyphosis, multilevel protruding discs, osteophytes and ossified lesions at disc area^1^. Using the Smith-Robinson approach, direct visualization and removal of disc herniation and osteophytes can be accomplished^1^. Success of this procedure depends on proper decompression of the neural structures and good bone fusion, which leads to the maintenance of the cervical lordosis and prevents recurrent spondylosis or neural compression^6^.

There are certain complications to multilevel ACDF: implant failure, nonunion and dysphagia, and these complications appeared to be greater in the elderly^2,7–14^. Moreover, the geriatric patients have higher comorbidity burden and a lower physiologic reserve for multiple organ systems, and greater odds to develop pulmonary complications, venous thromboembolism, urinary tract infection, cardiac complications and sepsis in 30 days^2,7,15^.

On the other hand, for well selected patients elder than 70, general practice had already established surgical intervention is safe and warranted to avoid permanent neurological impairment, to stabilize the disease process, and to result in some neurologic recovery^16–19^. To date, literature is few to guide the surgeons doing multilevel ACDF for patients with DCM over 70 years of age. The purposes of our study are:

1. To compare the clinical outcomes in multilevel ACDF in the elderly and younger cohorts.
2. To evaluate the complication profile between the elderly and younger cohorts in middle term follow-up period.

## Materials and methods

### Patient population

After the approval by the institutional review board, consecutive patients with degenerative cervical myelopathy treating by primary multilevel ACDF (3 or more levels) and plate fixation by a single surgeon from January 2006 to September 2019 were retrospectively reviewed. All research was performed in accordance with relevant guidelines and regulations, and informed consent was obtained from all participants or their legal guardians. The patients were divided into 2 groups.

1. Elderly group: aged 70 years old and above.
2. Younger group: aged below 70 years old.

**Figure 1:**
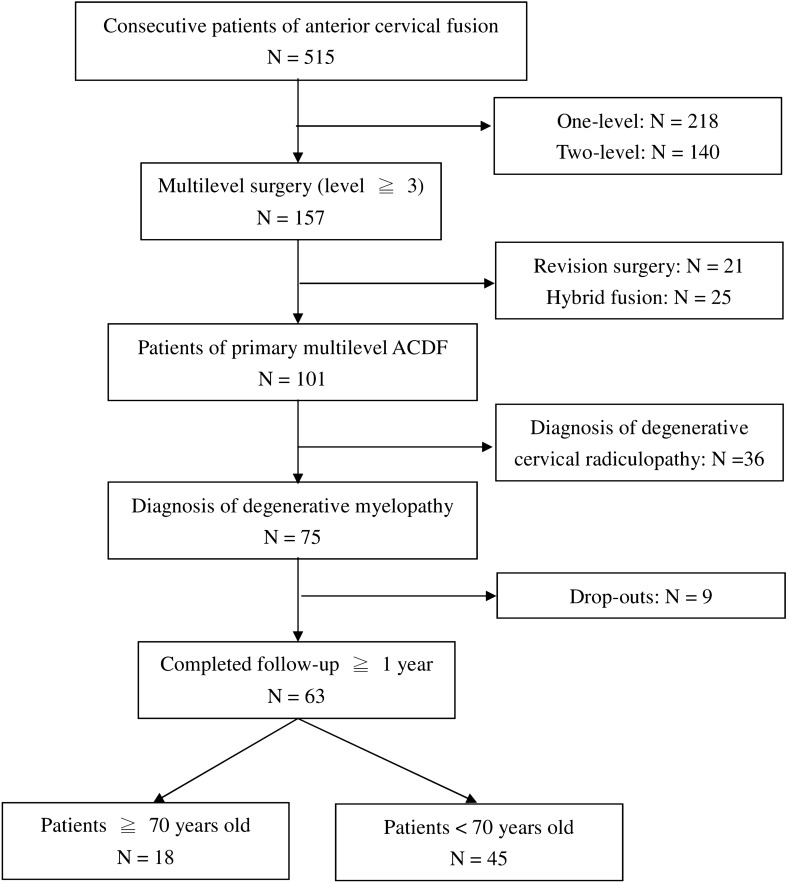
Flow diagram of numbers of individuals at each stage of study.

A total of 515 patients underwent ACDF of all levels were reviewed. Out of this, 101 patients underwent multilevel ACDF surgery in the study period were reviewed. The following patients were excluded in this study, patients being treated for cervical spine radiculopathy, cervical fractures, tumors, infections, and patients with previous cervical spine surgeries, and hybrid constructs. After exclusion, 69 patients diagnosed as degenerative cervical myelopathy were further reviewed and 63 out of them completed follow-up for a minimum of one year, were included in this study (Fig. 1). Clinical parameters such as age, gender, body mass index, American Society of Anesthesiologists (ASA) classification, social history, past medical history including Charlson comorbidity index were recorded at first visit. Surgical and hospitalization data including surgical level, estimated blood loss, skin-to-skin surgical duration, and length of hospital stay were collected.

### Surgical procedure

Surgeries were done by a single senior spine surgeon of the department. Patient was placed in a supine position with slight neck extension. After surface landmarks were carefully palpated, an appropriately sized longitudinal incision was made. A Smith–Robinson approach was used. Once proper exposure was done, surgical level was checked using X-ray. Caspar screws were placed to distract the disc space. Decompression via discectomy and removal of hypertrophic osteophytes were performed under microscope. Upper and lower endplates were prepared by curette to remove the cartilage and expose the subchondral bone after decompression was completed. By assuming every elderly as osteoporotic, a more delicate surgical skill is required to preserved the most endplate surface and osteoporotic bone underneath: 1. No burr even if efficient, 2. No use of angled curette tip. Use the side, 3. No “digging” bur “shaving”, 4. Be careful of balance uncovertebral resection, 5. Be careful of excessive posterior endplate decompression. Finally, a 3 mm central hole was made at every endplate surfaces needed to be fused to enhance bony fusion. Trial cage was placed with release of Caspar screw traction to assure its proper size and to avoid subsidence. Polyetheretherketone (PEEK) cages were selected and placed [Cervios cage (Synthes, Mathys Medical, Bettlach, Switzerland)] and filled with deep frozen allogeneic morselized cancellous graft. Portable X-ray was used to check the position of the PEEK cage. Lastly, an appropriately sized Reflex Hybrid plate system (Stryker, Allendale, NJ, USA) was applied. A Philadelphia cervical collar (Philadelphia Cervical Collar Co., Thorofare, NJ, USA) was used for six weeks in the post-operative period.

### Patient outcomes and radiographic evaluation

Pre- and post-operative subjective outcome measures assessed using the modified Odom’s criteria^10,20^. The result was determined as excellent, good, fair, and poor. The neurologic functional assessment was by the Japanese Orthopaedic Association (JOA) score, a 17-point scoring system to assess physical ability^21^. Patients were scored in the preoperative and postoperative 1 year. The recovery rate was calculated by Hirabayashi method (%) = (postoperative JOA score − preoperative JOA score) × 100/(17 − preoperative JOA score). Postoperative events were recorded including nonunion, cage subsidence, implant loosening, pseudoarthrosis, surgical site infection, adjacent segment pathology, revision surgery as surgical related and pneumonia, sepsis, kidney failure, cerebrovascular accident, myocardial infarction as medical disease related. These complications were grouped into short-term as occurrence within 1 year and middle-term as occurrence more than 1 year. The surgical complications were further categorized as need to have reoperation or not.

Patients’ anteroposterior and lateral radiographs were retrieved with the following time intervals: preoperative, immediate postoperative and postoperative 1 year and 2 years. The following parameters were assessed in lateral radiograph including the C2–C7 Cobb angle, C2–C7 sagittal vertical axis (SVA), and disc height of each level from C2 to T1. The C2–C7 Cobb angle was measured by drawing a parallel line to the superior end plate of C2 and another parallel line to the inferior end plate of C7. Perpendicular lines were drawn from these lines and the angle formed from their intersection is the Cobb angle. C2–C7 SVA was measured by drawing a plumb line from C2 body center and its shortest distance to posterosuperior corner of C7. Subsidence was defined as a decrease of ≥ 2.5 mm in disc heights in any of the operated levels^22^. Spinal fusion was determined on radiographs with the following parameters^23,24^: 1. Presence of bony trabeculae across the graft-host interface, 2. Bridging bone formation at either the anterior or posterior areas of the operated vertebrae, 3. Hazy interface between the cage and the endplates and lastly absence of lucencies around the cage, 4. No motion greater than 5 degrees on dynamic flexion/extension radiographs, 5. No change in interspinous process distance > 1 mm on dynamic flexion/extension radiographs. Implant stabilities were also assessed. MRI/CT evaluation was done only for those having worsening symptom, JOA score, or new neurologic deficit during follow-up period. All imaging studies obtained were blinded and reviewed by three board eligible orthopedic surgeons (C.A.L, A.S.L and C.C.N). If the judgement of fusion or stability was in doubt, meetings were held among C.A.L, A.S.L and C.C.N to address potential sources of bias and reach a final agreement.

### Statistical analysis

Data were input and analyzed using IBM SPSS Ver. 20.0 (IBM Corp., Armonk, NY, USA). Continuous variables were presented as means ± standard deviation. Comparisons were done using the Student’s t-test for analyzing continuous variables and the chi-square test and Fisher’s exact test whenever indicated for analyzing categorical variables. A two-tailed p-value of < 0.05 is considered statistically significant in all our analyses.

### Ethics approval

This study (Ref. No. 201901224B0) was approved by the institutional review board of Chang Gung Memorial Hospital, Linkou, Taiwan and appropriate informed consents were obtained.

## Results

A total of 63 patients including 45 males and 18 females were studied. Multilevel ACDF was performed at three-level in 49 patients, four-level in 13 patients and five-level in 1 patient. Total 204 cervical levels were operated on, and the most common levels were C5/6 with 63 segments (30.8%) and C4/5 with 61 segments (29.9%) operated on, followed by C3/4 with 42 segments (20.6%), C6/7 with 34 segments (16.7%) and lastly C7/T1 with 3 segments (1.5%) operated on. Eighteen patients were in the elderly group and 45 were in younger group. The demographic was tabulated in Table 1.

**Table 1:** Patients demographic.

	Elder group (n = 18)	Younger group (n = 45)	*p*-value
Age at Op	75 ± 4.3	60 ± 6.1	< 0.001*
Gender (male : female)	14 : 4	31 : 14	0.48
Level (3:4:5)	14 : 3 : 1	35 : 10 : 0	0.26
Pre-Op JOA score	11.6 ± 4.27	12.4 ± 2.44	0.44
Op time (minute)	298 ± 52.6	301 ± 41.9	0.82
Blood loss (milliliter)	56 ± 26.6	70 ± 88.7	0.53
Hospital stay (day)	4.9 ± 2.16	4.6 ± 1.10	0.47
ASA ≧ 3	94.4%	48.9%	< 0.001*
BMI	25.1 ± 7.27	26.6 ± 5.96	0.39
Smoker	6 (33.3%)	9 (20.0%)	0.52
Charlson comorbidity index	4.3 ± 1.03	2.1 ± 1.11	< 0.001*
Follow-up (month), range	43.8 ± 24.16 11.2–91.6	55.5 ± 44.26 12.3–168.9	0.29
**Comorbidities (n, %)**			
Hypertension	10 (55.6%)	24 (53.3%)	1
Diabetes	8 (44.4%)	11 (24.4%)	0.14
Liver disease	3 (16.7%)	3 (6.7%)	0.34
Peptic ulcer disease	3 (16.7%)	5 (11.1%)	0.68
Cerebral vascular accident	3 (16.7%)	2 (4.4%)	0.14
Chronic obstructive pulmonary disease	1 (5.6%)	2 (4.4%)	1
Solid tumor	1 (5.6%)	1 (2.2%)	0.49
Congestive heart failure	1 (5.6%)	1 (2.2%)	0.49

*Op time* skin-to-skin duration of operation, *ASA* American Society of Anesthesiologists Classification, *JOA score* Japanese Orthopaedic Association score, *BMI* body mass index.

Elder Group: age elder then 70; Younger group: age below 70.

*Statistical significance.

In the elderly group, 14 patients (78%) were men, with a mean age of 75 ± 4.3 (range: 70 ~ 87). The elderly group had Charlson comorbidity index of 4.3 ± 1.03 (3 ~ 7), BMI of 25.1 ± 7.27 kg/m^2^, and ASA III ratio 94.4%. In the younger group, 31 patients (69%) were men, with a mean age of 60 ± 6.1 (range: 43 ~ 70). The JOA score was 11.6 ± 4.27. The younger group had Charlson comorbidity index of 2.1 ± 1.11 (1 ~ 4), BMI of 26.6 ± 5.96 kg/m^2^, and ASA III ratio 48.9%. The JOA score was 12.4 ± 2.44. There are significant differences in the age (p < 0.001), Charlson comorbidity index (p < 0.001) and ASA III ratio (p < 0.001), but no significant difference between gender and BMI.

The elder group had 59 cervical levels with 14 three-level, 3 four-level and 1 five-level patients. The estimated blood loss was 56 cc (range 20 ~ 100). The surgical time was 298 min (219 ~ 378). The length of hospital stay was 4.9 days (range: 3–9), and follow-up period was 43.8 months (range: 12 ~ 92). In contrast, the younger group had 145 cervical levels with 35 three-level and 10 four-level patients. The estimated blood loss was 70 cc (20–500). The surgical time was 301 min. The length of hospital stay was 4.6 days (range: 3 ~ 7) and a follow-up period of 55.5 months (range: 12 ~ 169). There were no significant difference between groups in surgical level, estimated blood loss, surgical time, and length of hospital stay.

**Figure 2:**
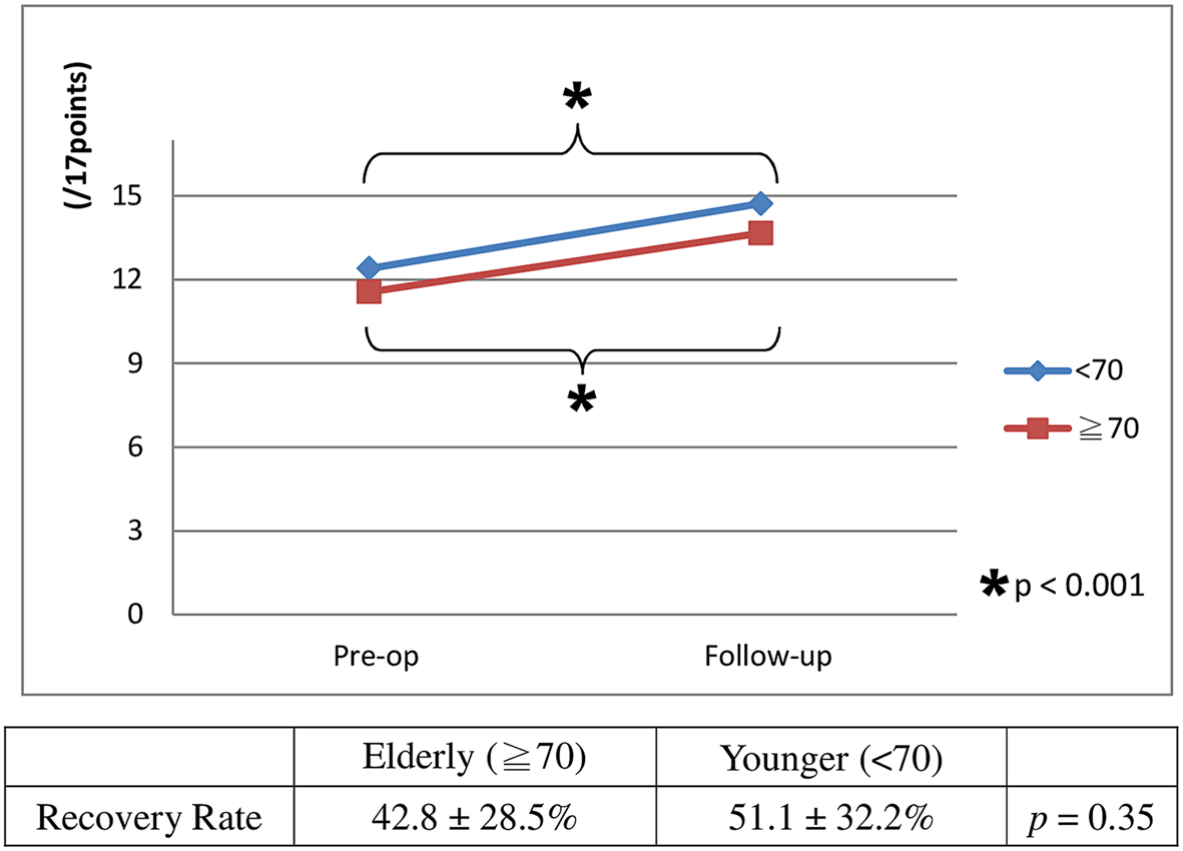
The clinical outcome of multilevel ACDF for degenerative cervical myelopathy as evaluated by Japanese Orthopaedic Association (JOA) score. The JOA score improved significantly in both groups.

As to clinical outcome, the JOA score improved to 13.7 ± 2.87 (range: 6 ~ 17) in the elderly group, and 14.7 ± 1.78 (range: 11 ~ 17) in the younger group at postoperative 1 year with statistical significance. The recovery rate was 42.8 ± 28.5% in the elderly group and 51.1 ± 32.2% in the younger group. (Fig. 2) In the elderly group, the modified Odom immediate postoperative scores were as follows, 11% excellent, 56% good, 28% fair and 5% patients rated poor. At postoperative one-year follow-up, 39% excellent, 28% good, 22% fair and 11% poor. In the younger group, the modified Odom immediate postoperative scores were as follows, 2% excellent, 64% good, 29% fair and 5% patients rated poor. At postoperative one-year follow-up, 20% excellent, 58% good, 18% fair and 4% poor. There was no significant difference between groups among JOA score, recovery rate and modified Odom score at immediate and one-year follow-up.

Table 2 showed short-term complications which were mostly surgical related. Two elderly patients (11.1%) needed reoperation. One patient had symptomatic screw loosening with dysphagia 1 month after index operation. He had removal of the loose screw. The other patient with diabetes mellitus and chronic kidney disease had deep surgical site infection at postoperative 1 year. He had debridement with removal of implant. These two patients then had recovered well thereafter. There were no durotomy, vertebral artery injury, epidural hematoma, esophageal injury, nor recurrent laryngeal nerve injury. For those surgical related complication not needing intervention, there were mostly cage subsidence, asymptomatic screw loosening, and transient dysphagia, hoarseness, nausea, and nasal congestion. The rate was 44.4% in the elderly and 37.8% in the younger group. For medical complication, 2 elderly patients and 1 younger patient had urinary tract infection developed in postoperative 1 month. Eleven patients (61.1%) in the elderly group experienced at least one short-term complication, compared with 16 patients (35.6%) in the younger group. There were no significant difference between groups except reoperation rate (p = 0.02).

**Table 2:** Short-term complications (within 1 year).

	Elder group (n = 18)	Younger group (n = 45)	*p*-value
**Surgical related (n, %)**	10 (55.6%)	16 (35.6%)	0.17
Reoperation required (n, %)	2 (11.1%)	0	0.02*
Symptomatic screw loosening	1 (5.5%)	0	
Deep surgical site infection	1 (5.5%)	0	
Reoperation not required (n, %)	8 (44.4%)	16 (35.6%)	0.57
Cage subsidence	3 (16.7%)	10 (24.4%)	
Asymptomatic screw loosening	2 (11.1%)	1 (2.2%)	
Dysphagia	1 (5.5%)	2 (4.4%)	
Hoarseness	1 (5.5%)	2 (4.4%)	
Nausea	1 (5.5%)	1 (2.2%)	
Nasal congestion	0	1 (2.2%)	
**Medical complication (n, %)**	2 (11.1%)	1 (2.2%)	0.19
Urinary tract infection	2 (11.1%)	1 (2.2%)	
**Total complication (n, %)**	11 (61.1%)	16 (35.6%)	0.09

*Statistical significance.

**Figure 3:**
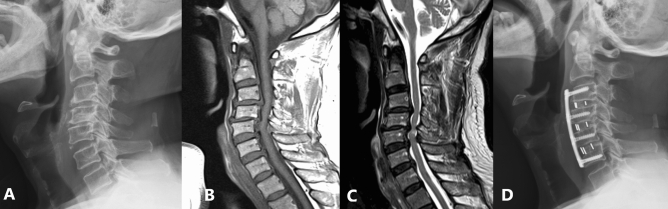
An 80-year-old male had degenerative cervical myelopathy from C3 to C6. Preoperative MRI T1W and T2W images shows spinal cord compression from C3 to C6 and cord edema at C4–C5. (**a**–**c**) He underwent three-level ACDF from C3 to C6 using four PEEK cages and one long anterior plate. Postoperative lateral cervical spine radiography at 1 year showed successful fusion from C3 to C6, maintenance of intervertebral heights, stable C2–C7 Cobb angle and improved C2–C7 SVA. (**d**) He had clinical improvement and smooth postoperative follow-up until 4 years later, having right middle cerebral artery territory infarction with left hemiparesis.

Table 3 showed middle-term complications. One elderly patient (5.6%) and 2 younger patients (4.4%) experienced radiographic nonunion. No reoperation was performed in the elderly group. Two reoperations were done in the younger group. One patient had C5–T1 ACDF and restenosis at index level noted at postoperative 5 years. He had subsequent cervical laminectomy for decompression. The other patient had C4–C7 ACDF and C2–C4 adjacent segment pathology noted at postoperative 14 years. He then had C2–C4 laminectomy and instrumentation. These patients had uneventful postoperative course thereafter. Medical complications occurred on 4 patients in the elderly group and 6 in the younger group. Two patients had deceased in the elderly group. One patient died of community-acquired pneumonia and sepsis at postoperative 6 years at the age of 86, the other patient died of acute myocardial infarction at postoperative 8 years at the age of 84. There were one with cerebrovascular accident at postoperative 4 year (Fig. 3) and one with kidney failure at postoperative 6 years. In the younger group, 3 patients had community-acquired pneumonia at postoperative 2, 5 and 5 years, 2 patients had cerebrovascular accident at postoperative 3 and 8 years, and 1 patient had kidney failure at postoperative 6 years. There was no significant difference between groups among surgical and medical middle-term complications.

**Table 3:** Middle-term complications (onset more than postoperative 1 year).

	Elder group (n = 18)	Younger group (n = 45)	*p*-value
**Surgical related (n, %)**	1 (5.6%)	4 (8.9%)	0.66
Reoperation required (n, %)	0	2 (4.4%)	0.36
Restenosis at index level	0	1 (2.2%)	
Adjacent segment pathology	0	1 (2.2%)	
Reoperation not required (n, %)	1 (5.6%)	2 (4.4%)	0.85
Radiographic nonunion	1 (5.6%)	2 (4.4%)	0.85
**Medical complication (n, %)**	4 (22.2%)	6 (13.3%)	0.38
Pneumonia	1^#^ (5.6%)	3 (6.7%)	
Myocardial infarction	1^#^ (5.6%)	0	
Cerebrovascular accident	1 (5.6%)	2 (4.4%)	
Kidney failure	1 (5.6%)	1 (2.2%)	
**Total complication (n, %)**	4 (22.2%)	9 (20.0%)	0.84

^#^Two mortalities: one patient died of pneumonia and sepsis at postoperative 6 years, the other patient died of acute myocardial infarction at postoperative 8 years.

The fusion rate was at 94.4% in the elderly and 95.5% in the younger group. The fusion time was 7.5 month in the elderly and 7.2 month in the younger group. For the radiographic parameters, in the elderly group, C2–C7 Cobb angle was 16.9 ± 13.31° preoperatively, 18.4 ± 11.12° at immediate postoperative period, and 16.7 ± 9.76° at postoperative two years. The C2–C7 SVA was 2.6 ± 2.22° preoperatively, 2.4 ± 1.44° at immediate postoperative period, and 2.4 ± 1.24° at postoperative two years. In the younger group, C2-C7 Cobb angle was 15.0 ± 14.12° preoperatively, 17.4 ± 10.83° at immediate postoperative period, and 16.5 ± 8.25° at postoperative 2 years. The C2–C7 SVA was 1.0 ± 1.01° preoperatively, 2.1 ± 1.45° at immediate postoperative period, and 1.4 ± 1.39° at postoperative two years. The disc heights of operative levels were maintained. There were statistical differences in C2–C7 SVA between the 2 groups both at preoperative period and at postoperative two years.

## Discussion

General practice had already established surgery is safe and effective for well selected patients elder than 70^16–19^. However, is surgery as extensive as multilevel ACDF safe enough? The current study is the first to report the clinical outcome of multilevel ACDF in the elderly more than 70 years of age and to compare the result with younger cohort. Our result showed the recovery ratio was 42.8% in the elderly group and 51.1% in the younger group. The modified Odom score showed approximately 65% patients experienced excellent/good outcome in postoperative one-year follow-up in both age groups. The clinical result was comparative to one long-term study of 3- and 4-level ACDF and another study of 4-level ACDF with a mean patients’ age of 50 s^1,10^. Interestingly, Isogai et al. found the recovery rate of 42% in geriatric patients more than 80 years of age when posterior decompressive surgery was performed^25^. Chen et al. found the recovery rate of 40.82% in patients more than 70 years of age when either approach was applied^26^. These authors attributed the reason to a lower preoperative JOA score in the older group, and concluded the therapeutic effects of surgery were significantly better for the young patients.

Multilevel ACDF helps to halt the debilitating neurologic impairment^2,5^, but however, surgeon may be dreadful of the medical risk especially this extensive surgery brought to the elderly patients. This medical complications were only reported in less-level ACDF in the elderly. Following one to two-level ACDF in patients above 80 years of age, there were 53.4% patients having at least one major medical complication in 90 days and 2.94% having mortality in 1 year^27^. Two nationwide database studies showed longer hospitalization and greater 30-day morbidity in patients more than 61^2^ and 75^7^ years of age. Even though the result of these 3 studies may not be applied to that of multilevel surgery, they did generate a concept that medical comorbidities including cardiovascular disease, hypertension, chronic pulmonary disease, chronic kidney disease and diabetes mellitus should be monitored in decision making^28,29^. Thus, we did not advocate operation in geriatric patient with high degree of preoperative comorbidities (Charlson comorbidity index ≧ 5) or with unstable condition, which was the same principle we adopt in our lumbar study^13^. In current cohort, the mean Charlson comorbidity index was 4.3 in elderly group. The mean length of hospital stay was comparable between the elderly and younger groups (4.9 and 4.6 days). The short-term medical complication involved only 2 elderly patients (11.1%) with urinary tract infection, who had severe myelopathy. No other short-term medical complications such as pulmonary, cardiac, or sepsis were recorded in the elderly group.

Surgical complications may stop the surgeon. Our study showed 55.6% surgical related complication. A significantly higher reoperation rate, 11.1%, was encountered in the elderly within one year. The most feared complication belongs to the osteoporosis entity including screw loosening and cage subsidence^13,14^. Larrata et al.^9^ reported 7% of the patients returned to surgery for hardware removal in multilevel study with a mean age of 55.9 years. Cage subsidence can be caused by a number of factors including female gender, oversized cage, and caudal levels in the surgical level^23,30^. The rate was reported to be 54.8% in female and 58.1% in multilevel surgery by Kao et al.^23^. In our study, the rates of cage subsidence were 16.7% in the elderly and 24.4% in the younger group. Despite the incidence rate fluctuated in the literature probably due to different patient demographic, cage subsidence was mostly a radiographic complication and did not affect postoperative alignment and neurologic improvement^23,30^. One special complication pertaining to multilevel ACDF was dysphagia, with a reported rate of 50% temporary and 10% permanent damage^20,31^. In one study temporary dysphagia was at 17.6% and transient hoarseness was at 11.8%^20^. In another study by Ebot et al.^32^, two (11%) patients with short-term dysphagia after 4-level ACDF were both female and more than 70 years of age, and it was concluded that age and gender were more predictive of developing dysphagia. In our study, about 5% of patients in both groups developed transient dysphagia and hoarseness. The low profile plate we used may result in the lower incidence of dysphagia^33,34^.

Middle term complications in our study revealed that comparable rate of surgical and medical complications occurred despite of different patient ages. For multilevel ACDF with more fusion interfaces, lower fusion rates and longer time to fusion were reported. Lee et al. found significantly longer time to fusion when compared with single-level ACDF and the mean periods were 4.09 and 5.25 months for 3-level and 4-level ACDF respectively^35^. Wewel et al. also evaluated fusion of 3- and 4-level ACDF and found 42% and 56% pseudarthrosis rate respectively^36^. On the contrary, the fusion rate studied by Garza-Ramos et al. was 84.6% after 4-level and 94.4% after 3-level ACDF^10^. The fusion rate after 4-level ACDF was 94% by Wang et al.’s study^1^. In multilevel ACDF for the elderly, we believed that a more delicate surgical skill is required to preserve the most endplate surface and osteoporotic bone underneath. The fusion rate in our study was 94.4% for the elderly and 95.5% for the younger. No patients experienced symptomatic pseudarthrosis.

While we evaluated multilevel ACDF, optimal surgical strategy for the geriatric is still controversial^1,3^. A multicenter prospective study by Inose et al.^37^ revealed no significant differences in clinical outcomes between approaches, but cervical alignment worsened after posterior surgery. In the present study, we demonstrated comparable length of hospital stays between the elderly and young groups. Our rationale was multilevel ACDF was associated with less postoperative neck pain, less wound sufferance and better cervical alignment maintenance. Thus, earlier function recovery would ensue, even in the elderly group.

There were several limitations to this study. First, it was retrospective with a small number of patients. However, the current study is the first to report the effectiveness and complication of multilevel ACDF in the elderly with DCM by single experienced surgeon. Second, the patient’s pain scale and quality of life scale were not recorded. These data represent the clinical outcome in a more comprehensive way. On the other hand, we did report a very detailed complication profile than others and hoped to help the elderly who are willing and need to have multilevel ACDF. Third, osteoporosis was not assessed routinely by dual-energy x-ray absorptiometry. Nevertheless, we did feel the bone quality during surgery and treated every patient with a more delicate surgical skill by assuming they were osteoporotic.

## Conclusion

Multilevel ACDF surgery is a feasible option to provide symptomatic relief and neurologic improvement for elderly myelopathic patients suffering from multilevel cervical degenerative disease. It showed comparative outcome with younger cohort. Despite the extensiveness of the surgery, length of hospital stay is not adversely affected. The elderly patients in this study have shown immediate and maintained improvement of their symptoms with less neck pain. Satisfactory fusion rate with less graft or implant complication could be achieved in the osteoporotic setting of these elder patients. For the geriatric patient with DCM, there is a trend of lower preoperative JOA score and recovery rate.

## Abbreviations

ACDF: Anterior cervical discectomy and fusion
JOA score: Japanese orthopaedic association score
BMI: Body mass index
ASA: American society of anesthesiologists classification
DCM: Degenerative cervical myelopathy
PEEK: Polyetheretherketone
SVA: Sagittal vertical axis
